# Phosphorylation of OCT4 Serine 236 Inhibits Germ Cell Tumor Growth by Inducing Differentiation

**DOI:** 10.3390/cancers12092601

**Published:** 2020-09-11

**Authors:** Dong Keon Kim, Bomin Song, Suji Han, Hansol Jang, Seung-Hyun Bae, Hee Yeon Kim, Seon-Hyeong Lee, Seungjin Lee, Jong Kwang Kim, Han-Seong Kim, Kyeong-Man Hong, Byung Il Lee, Hong-Duk Youn, Soo-Youl Kim, Sang Won Kang, Hyonchol Jang

**Affiliations:** 1 Research Institute, National Cancer Center, Goyang 10408, Korea; fastfly84@naver.com (D.K.K.); bominsong0625@gmail.com (B.S.); hanbonoboss@ncc.re.kr (S.H.); 97135@ncc.re.kr (H.J.); 97119@ncc.re.kr (S.-H.B.); 74790@ncc.re.kr (H.Y.K.); shlee1987@gmail.com (S.-H.L.); 97143@ncc.re.kr (S.L.); jk@ncc.re.kr (J.K.K.); kmhong@ncc.re.kr (K.-M.H.); bilee@ncc.re.kr (B.I.L.); kimsooyoul@gmail.com (S.-Y.K.); 2 Department of Life Science, Ewha Womans University, Seoul 03760, Korea; kangsw@ewha.ac.kr; 3 Department of Cancer Biomedical Science, National Cancer Center Graduate School of Cancer Science and Policy, Goyang 10408, Korea; 4 Department of Pathology, Inje University Ilsan Paik Hospital, Goyang 10308, Korea; hskim@paik.ac.kr; 5 National Creative Research Center for Epigenome Reprogramming Network, Department of Biomedical Sciences, Ischemic/Hypoxic Disease Institute, Seoul National University College of Medicine, Seoul 03080; Korea; hdyoun@snu.ac.kr

**Keywords:** Oct4, phosphorylation, germ cell tumor, cancer differentiation, OCT4 serine 236, OCT4 inhibitor

## Abstract

**Simple Summary:**

Octamer-binding transcription factor 4 (OCT4) plays an important role in early embryonic development, but is rarely expressed in adults. However, in many cancer cells, this gene is re-expressed, making the cancer malignant. This present study revealed that inhibiting OCT4 transcriptional activity induces cancer cell differentiation and growth retardation. Specifically, when the phosphorylation of OCT4 serine 236 increases by interfering with the binding of protein phosphatase 1 (PP1) to OCT4, OCT4 loses its transcriptional activity and cancer cells differentiate. Therefore, this study presents the basis for the development of protein-protein interaction inhibitors that inhibit the binding of OCT4 and PP1 for cancer treatment.

**Abstract:**

Octamer-binding transcription factor 4 (Oct4) plays an important role in maintaining pluripotency in embryonic stem cells and is closely related to the malignancies of various cancers. Although posttranslational modifications of Oct4 have been widely studied, most of these have not yet been fully characterized, especially in cancer. In this study, we investigated the role of phosphorylation of serine 236 of OCT4 [OCT4 (S236)] in human germ cell tumors (GCTs). OCT4 was phosphorylated at S236 in a cell cycle-dependent manner in a patient sample and GCT cell lines. The substitution of endogenous OCT4 by a mimic of phosphorylated OCT4 with a serine-to-aspartate mutation at S236 (S236D) resulted in tumor cell differentiation, growth retardation, and inhibition of tumor sphere formation. GCT cells expressing OCT4 S236D instead of endogenous OCT4 were similar to cells with OCT4 depletion at the mRNA transcript level as well as in the phenotype. OCT4 S236D also induced tumor cell differentiation and growth retardation in mouse xenograft experiments. Inhibition of protein phosphatase 1 by chemicals or short hairpin RNAs increased phosphorylation at OCT4 (S236) and resulted in the differentiation of GCTs. These results reveal the role of OCT4 (S236) phosphorylation in GCTs and suggest a new strategy for suppressing OCT4 in cancer.

## 1. Introduction

Octamer-binding transcription factor 4 (Oct4; encoded by Pou5f1) is a crucial factor during mammalian development [1] for maintaining pluripotency and the self-renewal of embryonic stem cells (ESCs) [2,3,4]. Among the variants of human OCT4, only OCT4A, referred to as OCT4 in most reports [5] and herein, can maintain ESC self-renewal. OCT4 is also expressed in a variety of solid tumors, particularly in small populations (or cancer stem cells) [6], and is closely related to cancer malignancies [7,8,9,10,11,12,13,14]. In addition, a recent Pan-Cancer Atlas project revealed that OCT4 is a crucial factor in constructing a stemness index that quantifies the degree of stemness, which is closely related to cancer metastasis and patient prognosis [15]. Due to the importance of Oct4, many studies have examined the regulation of Oct4 at the transcriptional [16,17,18,19] and posttranslational levels [4,20,21,22,23].

Since the protein level of Oct4 does not sufficiently represent its transcriptional activity [4], posttranslational modifications (PTMs) of Oct4 are very important. Various PTMs, such as sumoylation, ubiquitination, O-GlcNAcylation, methylation, acetylation, and phosphorylation, have been reported in lysine (K), serine (S), threonine (T), and arginine residues of Oct4 (www.phosphosite.org). The functions of several PTMs have been reported, but most are not yet fully characterized, especially in cancer. For example, ubiquitination of OCT4 decreases OCT4 protein stability in human ESCs [24,25], and O-GlcNAcylation of murine OCT4 at T228 is important for the maintenance and acquisition of pluripotency in mouse ESCs [4], but their functions in cancer have not yet been determined. In cases of acetylation [26], methylation, and sumoylation [27,28], their functions remain unclear, while the phosphorylation of OCT4 may play multiple or opposite roles depending on the residue where it occurs. Phosphorylation of OCT4 at S12 [OCT4 (S12)] is important for its interaction with Pin1 and increases the transcriptional activity [29], whereas phosphorylation of OCT4(S111) by extracellular signal-regulated kinases 1 and 2 reduces its activity by increasing ubiquitination and degradation [30]. Phosphorylation of OCT4 (T235) by AKT increases its stability [22]; however, another study showed that phosphorylation at the corresponding residue of murine Oct4 at T228 accelerated its degradation [31]. In mouse ESCs, phosphorylation of murine Oct4(T343) is important for OCT4 activity [32], but phosphorylation of Oct4 (S347) by c-Jun N-terminal kinases decreases its protein stability [33].

The phosphorylation of murine Oct4 (S229) is one of the best studied PTMs of Oct4. In mouse ESCs, Aurora kinase b phosphorylates Oct4 (S229) during the G2/M phase to induce the dissociation of Oct4 from chromatin, whereas protein phosphatase 1 (PP1) dephosphorylates Oct4 at the same residue during M/G1 transition [34]. This cell cycle-dependent Oct4 phosphorylation is elaborately regulated by cyclin-dependent kinase 1, through which the cell cycle dynamically regulates the pluripotency transcription program [35]. Although phosphorylation of human OCT4 (S236) (corresponding to murine S229) has also been detected by mass spectrometry in human ESCs [36], the role of phosphorylated OCT4(S236) [p-OCT4(S236)] in human cancer has not yet been revealed.

Here, we focused on the role of p-OCT4 (S236) in human cancer. Germ cell tumor (GCT) cells were chosen as a model system to study PTMs of OCT4 because all GCT cells were positive for OCT4 expression in vitro [37]. Notably, OCT4 is expressed only in small cell populations in most types of cancer in vitro [6]. GCTs, which commonly occur in the gonads and are one of the most common malignant tumors in young men and women [37], are undifferentiated and maintain pluripotency in a manner similar to ESCs [37,38]. In addition, OCT4 expression is highest in testicular GCTs [15], and OCT4 plays a critical role in regulating the malignant potential of GCTs [37,39]; thus, GCTs are suitable for studying the role of PTMs of OCT4.

## 2. Results

### 2.1. OCT4 Is Phosphorylated at S236 in a Cell Cycle-Dependent Manner in Human Cancer Cells

**Figure 1:**
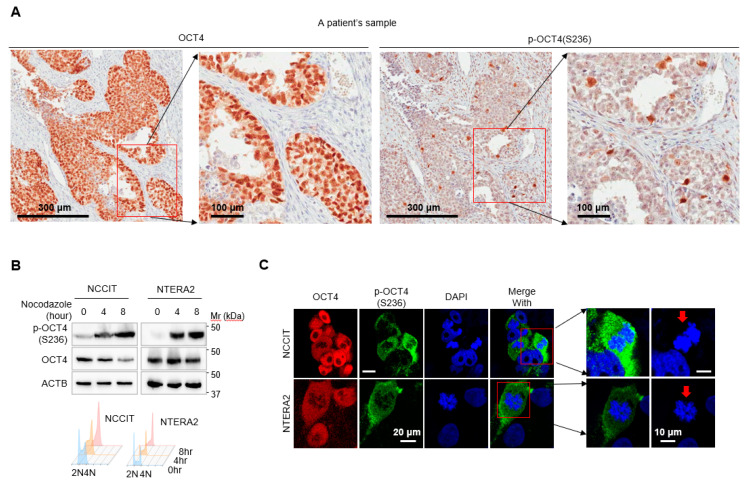
Octamer-binding transcription factor 4 (OCT4) is phosphorylated at S236 in a cell cycle-dependent manner in human cancer cells. (**A**) Immunohistochemistry of a GCT patient sample by the indicated antibodies. p-OCT4 (S236): phosphorylated OCT4 at S236. (**B**) NCCIT and NTERA-2 cells were stained with propidium iodide after treatment with nocodazole (50 ng/mL) for the indicated times. The levels of OCT4 and p-OCT4 (S236) were determined by Western blot (Top). Simultaneously, the DNA contents of individual cells were analyzed by flow cytometry (FACSVerse; BD Biosciences). Histogram images were obtained with FlowJo software (bottom). (**C**) NCCIT and NTERA-2 cells were immunostained with the indicated antibodies. Confocal images were obtained using an LSM 510 confocal microscope (Carl Zeiss). On the right side, larger images for cells with condensed chromatin (arrows) were shown.

We investigated the role of OCT4 phosphorylation at S236, which corresponds to S229 of murine Oct4, in human cancer. To this end, we first investigated whether p-OCT4 (S236) was detected in a human cancer patient sample. A sample from a testicular GCT patient was tested by immunohistochemistry using anti-OCT4 and anti-p-OCT4 (S236) antibodies. OCT4 was specifically detected in most of the tumor cells, and p-OCT4 (S236) was detected in a small portion of tumor cells (Figure 1A). Since OCT4 was only phosphorylated at S229 in the G2/M phase of the cell cycle in mouse ESCs [34,35] and only a small portion of OCT4-positive tumor cells were positive for p-OCT4(S236) in a GCT sample (Figure 1A), we inferred that the phosphorylation of OCT4(S236) in human cancer cells would also occur at the G2/M phase. 

Two GCT cell lines, NCCIT and NTERA-2, were arrested in the G2/M phase by treatment with nocodazole, and the levels of p-OCT4 (S236) and OCT4 were evaluated by Western blot. The level of p-OCT4 (S236) was apparently increased in the G2/M phase, while the level of OCT4 was not (Figure 1B, top). Propidium iodide staining and flow cytometry analysis confirmed that nocodazole treatment shifted the cell cycle into the G2/M phase in a time-dependent manner (Figure 1B, bottom). Immunofluorescence staining and confocal microscopy revealed that all cells were positive for OCT4, whereas only small numbers of cells were positive for p-OCT4(S236) in the NCCIT and NTERA-2 cell lines (Figure 1C, left). All cells positive for p-OCT4 (S236) showed condensed nuclei (Figure 1C, right), suggesting that p-OCT4(S236) occurred in the mitotic phase. Collectively, these results indicate that OCT4 is phosphorylated at S236 in a cell cycle-dependent manner in human cancer, similarly to mouse ESCs.

### 2.2. Replacement of Endogenous OCT4 with a Mimic of p-OCT4(S236) Causes a Phenotype Similar to OCT4 Depletion in GCTs

**Figure 2:**
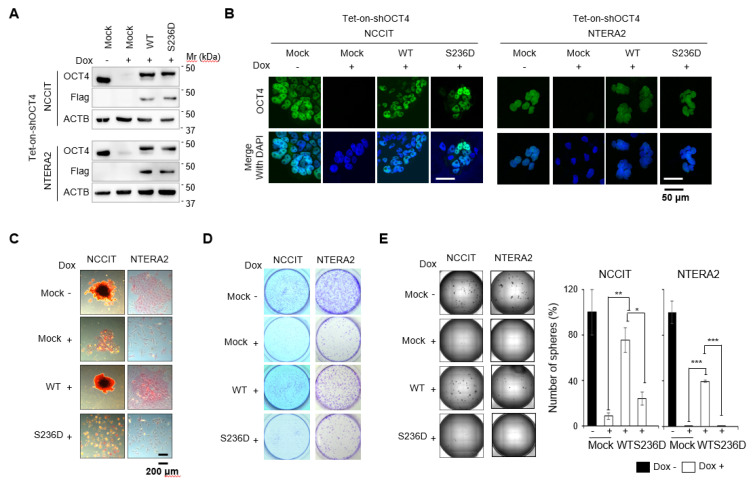
Replacement of endogenous OCT4 with a mimic of p-OCT4 (S236) causes a phenotype similar to OCT4 depletion in GCTs. (**A**) Generation of cancer cell lines expressing Flag-tagged WT and phospho-mimetic mutant OCT4 instead of endogenous OCT4. NCCIT and NTERA-2 cells stably incorporating Tet-on-shOCT4 were then stably incorporated with a WT and a phospho-mimetic (S236D) OCT4 expression vector. The stable incorporation of the empty vector (Mock) was used as the control. After treatment with Dox (1 µg/mL) for four days for NCCIT and seven days for NTERA-2, knockdown of endogenous OCT4 and rescue with OCT4 WT and S236D were confirmed by Western blot. (**B**) The generated cells were immunostained with anti-OCT4 antibody and analyzed by confocal microscopy. (**C**,**D**) The indicated cells were seeded at a low density and stained with AP or crystal violet at seven or 14 days after seeding, respectively. Representative images from three independent experiments are shown. (**E**) Single-cell suspensions of the indicated cell lines were plated onto ultralow attachment plates. Two weeks after plating, the entire images of spheres in 96-well cell culture plates were obtained with Cytation 3 (BioTek, Winooski, VT, USA). The relative number of spheres (% ± SD, N = 3), compared to that from doxycycline-untreated mock cells, was analyzed. * p < 0.05, ** p < 0.01, *** p < 0.001.

Next, we investigated whether p-OCT4 (S236) plays a role in human cancer. NCCIT and NTERA-2 cell lines expressing a mimic of p-OCT4 (S236) instead of endogenous OCT4 were generated. In these cell lines, endogenous OCT4 was depleted by the doxycycline (Dox)-dependent expression of small hairpin RNA (shRNA) against OCT4, and then the cells were rescued with exogenous Flag-tagged OCT4 wild type (WT) or mutant (serine to aspartate mutation at S236; S236D). Western blot and immunofluorescence staining showed that these cell lines were successfully prepared (Figure 2A,B). Importantly, the expression levels of endogenous OCT4 and Flag-tagged OCT4 WT and S236D were similar (Figure 2A,B); therefore, the role of OCT4 phosphorylation could be studied under physiological conditions. The level of OCT4 protein expression is very important for its function [40], and different levels of OCT4 expression can lead to additional functional differences. Treatment with Dox results in OCT4 depletion and induces GCT differentiation [37]. The empty vector rescue (Mock) did not inhibit the reduction of alkaline phosphatase (AP) staining, a marker of undifferentiation (Figure 2C). The rescue with OCT4 WT almost completely suppressed the reduction of AP staining by Dox treatment, whereas the rescue with OCT4 S236D did not (Figure 2C). The clonogenic assay showed that OCT4 WT-rescued cells proliferated similarly to non-OCT4-depleted cells, whereas OCT4 S236D-rescued cells did not proliferate well, similar to OCT4-depleted cells (Figure 2D). In addition, OCT4 S236D-rescued cells did not form tumor spheres, similar to OCT4-depleted cells, whereas OCT4 WT-rescued cells showed a tumor sphere formation comparable to non-OCT4-depleted cells (Figure 2E). These results suggest that the substitution of OCT4 with p-OCT4(S236) results in a phenotype similar to OCT4 depletion in GCTs.

### 2.3. Cells Expressing a Mimic of p-OCT4(S236) Instead of Endogenous OCT4 are Similar to Cells with OCT4 Depletion at the mRNA Transcript Level

**Figure 3:**
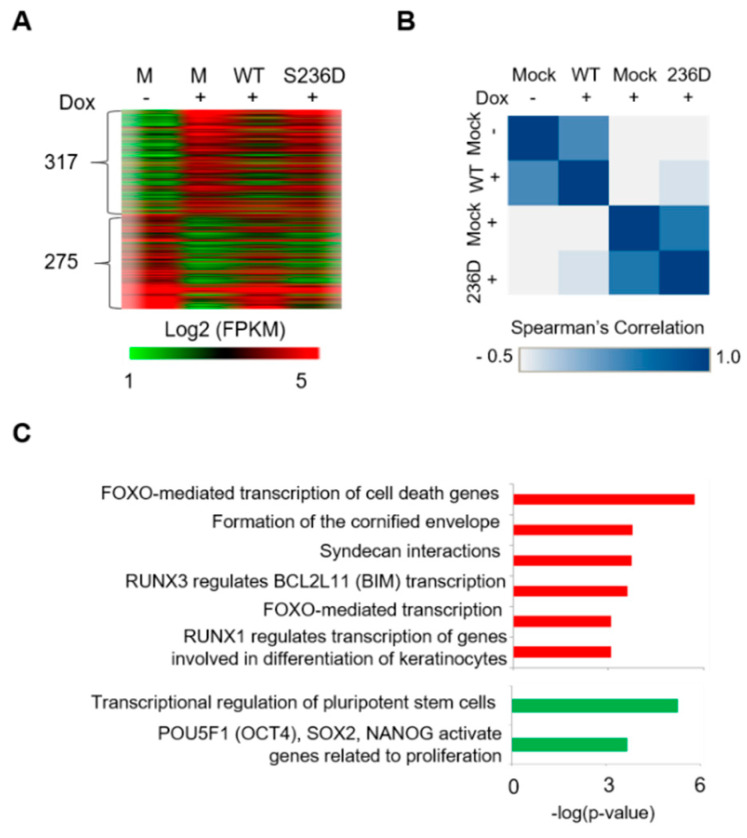
Cells expressing a mimic of p-OCT4 (S236) instead of endogenous OCT4 are similar to cells with OCT4 depletion at the mRNA transcript level. (**A**) Total RNA extracted from NCCIT cell lines harboring endogenous OCT4, depleted OCT4, OCT4 WT rescue, and OCT4 S236D rescue were sequenced, and DEGs among the samples were analyzed. Genes whose expression changed more than two-fold are shown. The heat map was generated using the Multi-Experiment Viewer 4.9 software. The gene list is presented in Table S1. (**B**) Spearman’s rho correlation coefficients for all pairs of samples. (**C**) DEGs between OCT4 WT and S236D were classified by Reactome analysis (reactome.org). Results with a *p*-value < 10^−3^ are shown. Compared to the WT, the genes upregulated (red) and downregulated (green) in S236D are colored.

To investigate the effect of p-OCT4 (S236) on the transcription of GCTs, mRNA was analyzed by RNA sequencing (RNA-Seq) in genetically modified NCCIT cells (control: Mock, Dox −; OCT4-depleted: Mock, Dox +; OCT4 WT-rescued: WT, Dox +; OCT4 S236D-rescued: S236D, Dox +). When OCT4 was depleted, 592 genes changed more than two-fold (Figure 3A). Of the 317 genes upregulated by OCT4 depletion, most were downregulated by the OCT4 WT rescue but not by the OCT4 S236D rescue (Figure 3A). Of the 275 genes downregulated by OCT4 depletion, most were upregulated by the OCT4 WT rescue but not by the OCT4 S236D rescue (Figure 3A, Supplementary Table S1). Spearman’s rank-correlation showed that OCT4-depleted cells and OCT4 S236D-rescued cells had similar mRNA expression patterns (Figure 3B).

The gene ontology analysis of differentially expressed genes (DEGs) between OCT4 WT- and S236D-rescued cells using a Reactome analysis showed that, with OCT4 S236D, cell death and differentiation-related genes were upregulated and stemness maintenance-related genes were downregulated (Figure 3C). These results suggest that the substitution of OCT4 with p-OCT4 (S236) results in a change similar to OCT4 depletion at the mRNA transcript level in GCTs.

### 2.4. GCT Cells Expressing a Mimic of p-OCT4(S236) instead of Endogenous OCT4 Exhibit Reduced growth and Increased Differentiation in Vivo

**Figure 4:**
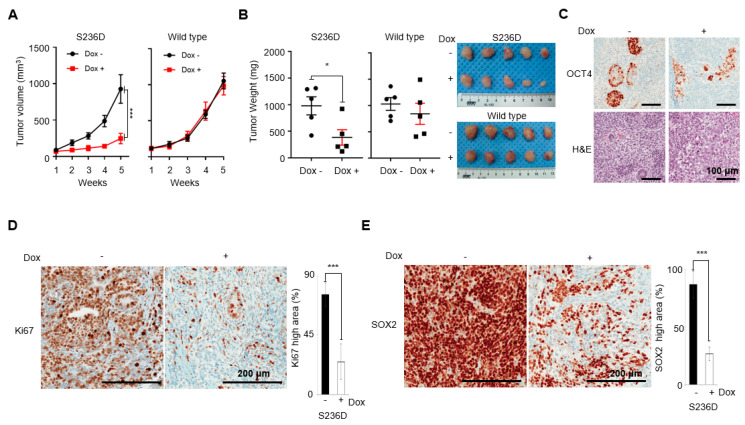
GCT cells expressing a mimic of p-OCT4 (S236) instead of endogenous OCT4 exhibit reduced growth and increased differentiation in vivo. (**A**) Difference in xenograft volumes between WT and S236D tumor. When the volume of the tumor mass reached 50–100 mm^3^, five mice were randomly selected for doxycycline induction with the rest for the vehicle-treated control group. With tumor size being measured for five weeks, doxycycline-treated xenografts showed significantly lower tumor volumes (N = 5, *** p < 0.001). (**B**) Final weight of tumor mass between WT and S236D. Graphs represent the mean ± standard error of the mean (*n* = 5; * *p* < 0.05). Images of tumors are shown on the right. (**C**) Immunohistochemistry with anti-OCT4 antibody indicates decreased OCT4 expression in Dox-treated samples. H&E: hematoxylin and eosin. (**D**) Ki67 staining shows that the proliferation is reduced in Dox-treated S236D. The degree of Ki67 staining was quantified by calculating the areas showing more than 50% Ki-67-positive cell percentage within xenograft explants. *** p < 0.001 relative to Dox nontreated S236D. (**E**) Reduced SOX2 expression in Dox-treated S236D. The degree of SOX2 staining was quantified by calculating the areas showing strong SOX2-positivity within xenograft explants. *** p < 0.001 relative to Dox nontreated S236D.

Since OCT4 S236D mimicked OCT4 depletion at the functional and transcriptional levels in vitro, we next investigated the effect of OCT4 S236D in vivo. NTERA-2 Tet-on-shOCT4 cell lines rescued with OCT4 S236D or WT were implanted into the flanks of nude mice. When the tumors were tangible, mice were grouped randomly, and vehicle or Dox was administered. In cases of OCT4 S236D rescue, Dox administration significantly reduced the tumor volume and weight compared to the vehicle administration group, while there were no significant changes in OCT4 WT rescue (Figure 4A,B). Thus, mouse xenograft data suggest again the important roles of p-OCT4 (S236) in the proliferation of GCTs. 

The immunohistochemistry of a xenograft with an anti-OCT4 antibody showed that Dox reduced OCT4 levels (probably by depleting endogenous OCT4) as intended (Figure 4C). In cases of OCT4 S236D rescue, the Dox-administered group showed a significant reduction in Ki67 staining, a marker of proliferating cells, when compared to the vehicle-administered group; the reduction in the tumor size and volume was primarily due to growth retardation (Figure 4D). Immunohistochemistry using anti-SOX2 antibody, an undifferentiation marker, showed that the Dox-administered OCT4 S236D group had significantly fewer SOX2-positive cells compared to the vehicle-administered group (Figure 4E). These results suggest that substitution of OCT4 with p-OCT4 (S236) results in GCT differentiation and growth inhibition in vivo, which is consistent with our in vitro results.

### 2.5. An Increase in p-OCT4(S236) by Inhibition of PP1 Results in Differentiation of GCTs

**Figure 5:**
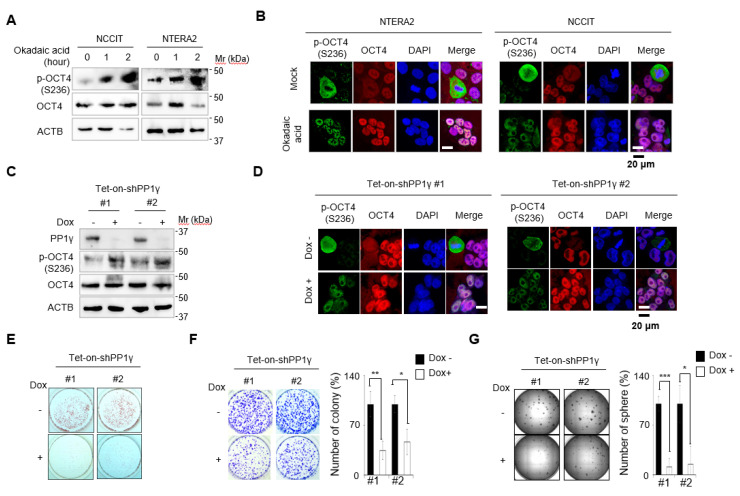
Inhibition of PP1 results in accumulation of p-OCT4(S236) and differentiation of GCTs. (**A**) NCCIT and NTERA-2 cells were treated with okadaic acid (50 nM), an inhibitor of PP1, for the indicated times, and the levels of OCT4 and p-OCT4(S236) were determined by Western blot. (**B**) Cells treated with okadaic acid (50 nM) for 2 h were immunostained with the indicated antibodies and analyzed by confocal microscopy. (**C**) NCCIT cells stably incorporating Tet-on-shPP1γ #1 and #2 were treated with or without Dox for four days. PP1γ, OCT4, and p-OCT4 (S236) levels were measured by Western blot. (**D**) NCCIT cells indicated in Figure 5C were immunostained with the indicated antibodies and analyzed by confocal microscopy. (**E**,**F**) Cells were seeded at a low density with or without Dox treatment. Cells were stained with (**E**) AP or (**F**) crystal violet at seven or 14 days after seeding, respectively. (**G**) Cells were plated for tumor sphere formation with or without Dox treatment. Two weeks after plating, the entire images of spheres in 96-well cell culture plates were obtained using Cytation 3. The relative number of colonies (%) for (**F**) and spheres (%) for (**G**) is indicated as the mean ± standard deviation (N = 3, * p < 0.05, ** p < 0.01, *** p < 0.001 relative to Dox nontreated cells), and representative images from three independent experiments are shown.

As the substitution of endogenous OCT4 with a mimic of p-OCT4 (S236) affected GCT differentiation and growth, we investigated whether the increase in actual p-OCT4 (S236) had a similar effect. Based on reports that PP1 dephosphorylates murine Oct4(S229) in mouse ESCs [34,35], we investigated whether PP1 also dephosphorylates human OCT4(S236) in GCTs. Western blot analysis revealed that the inhibition of PP1 by treatment with okadaic acid, a broad inhibitor of PP1 and PP2A, resulted in the accumulation of p-OCT4(S236) in a time-dependent manner (Figure 5A). Immunofluorescence staining and confocal microscopy showed that only cells with condensed nuclei in NTERA-2 and NCCIT cell lines were positive for p-OCT4(S236), but all cells became positive for p-OCT4(S236) when treated with okadaic acid (Figure 5B). In addition, p-OCT4 (S236) was found in nuclei (Figure 5B), suggesting that phosphorylation of OCT4 (S236) did not alter the intracellular localization of OCT4. 

Knockdown of PP1γ, a catalytic subunit of PP1 complex, by the Dox-dependent expression of two different shRNAs also resulted in an increase in p-OCT4(S236), as confirmed by Western blot and confocal microscopy (Figure 5C,D). The increase in p-OCT4 (S236) by the Dox-dependent expression of shRNAs against PP1γ resulted in an apparent decrease of AP-positive colonies (Figure 5E), a significant decrease in proliferation by clonogenic assay (Figure 5F), and an apparent decrease of the tumor sphere formation (Figure 5G). These data suggest that the induction of p-OCT4 (S236) by inhibition of PP1 induces growth inhibition and differentiation of GCTs.

## 3. Discussion

Although p-OCT4 (S236) was detected in human ESCs [36] and the role of the corresponding p-Oct4 (S229) in mouse ESCs was studied [34,35], its presence and role in human cancers have yet to be elucidated. In this study, we demonstrate that p-OCT4 (S236) was present in human cancer by showing that p-OCT4 (S236) was detected in a human GCT patient sample (Figure 1A) and human GCT cell-lines (Figure 1B,C). The specificity of the anti-p-OCT4 (S236) antibody has been confirmed in mouse ESCs [34], and since the sequence of the epitope for the antibody is the same in humans and mice, it is thought to have the same specificity in humans. In a GCT patient sample, only a small portion of OCT4 was phosphorylated at S236 (Figure 1A), which can be understood through the discovery that p-OCT4 (S236) was only detected in the mitotic phase in GCT cell-lines (Figure 1B,C). Consistent with this idea, the immunohistochemistry of the same patient sample with CKAP2 (Figure S1), which specifically detects cells in the mitotic phase [41], showed that the proportion of CKAP2-positive cells was similar to that of p-OCT4 (S236) positive cells. One limitation of this study was that only one patient sample was used, and further studies are needed to determine the frequency of detection of p-OCT4 (S236) in human cancer. In this study, we also demonstrated the function of p-OCT4 (S236) in GCT. OCT4 knockdown has induced GCT growth retardation and differentiation in vitro [37]. Replacing endogenous OCT4 with a mimic of p-OCT4(S236) or artificially increasing p-OCT4(S236) by inhibiting PP1 resulted in a phenotype similar to OCT4 depletion in GCTs in vitro (Figure 2 and Figure 5). In addition, replacing endogenous OCT4 with OCT4 S236D reduced tumor growth and a population of cells positive for SOX2, one of the undifferentiation markers, in mouse xenograft experiments in vivo (Figure 4). These data suggest that p-OCT4 (S236) inhibits GCT growth by hampering OCT4 activity, thereby inducing differentiation.

More than 40 PTMs of Oct4 have been identified (www.phosphosite.org), but most of their functions have not yet been investigated. Given that the Oct4 protein level does not sufficiently represent its transcriptional activity [4], understanding the function of each PTM is essential to fully elucidating the function of Oct4. One obstacle to understanding the functions of Oct4 PTMs is the inconsistent reports on the function of the same PTM. Sumoylation of OCT4 (K118 for mice and K123 for humans) affected protein stability positively in mice but negatively in humans [27,28]. Phosphorylation of OCT4 (T258) for humans and OCT4 (T228) for mice by Akt affected Oct4 activity positively in humans but negatively in mice [22,31]. The difference between species may be one reason, but differences in Oct4 protein expression levels may be more important. Oct4 protein expression above or below a certain level induces ESC differentiation [40]. This means that the expression level of the PTM mimic or OCT4-defective mutants introduced in the experimental process can affect the cell phenotype separately from the PTM. Therefore, this study attempted to accurately investigate the role of OCT4 by removing endogenous OCT4 and expressing similar levels of OCT4 PTM mutants (Figure 2A). The RNA-Seq results showed that OCT4 WT-rescued cells were similar at the mRNA transcript level to WT cells (Figure 3B), indicating that the OCT4 expression levels in our experimental setup did not affect cell physiology. In this study, the function of p-OCT4 (S236) in human GCTs was similar to that of p-Oct4 (S229) in mouse ESCs [34,35]. Phosphorylation of OCT4 (S236) occurred in a cell-cycle dependent manner (Figure 1), and PP1 was responsible for the dephosphorylation of the site (Figure 5). p-OCT4 (S236) lost transcriptional activity (Figure 3) and could not maintain stemness (Figure 2). These results suggest that phosphorylation of OCT4 (S236) regulates the binding of OCT4 to chromatin in the mitotic phase in both mice and humans.

**Figure 6:**
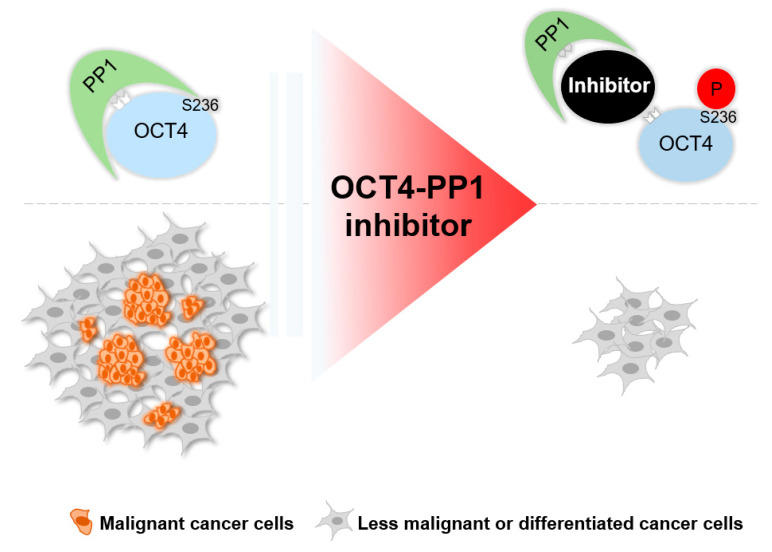
Potential strategy model to inhibit OCT4–PP1 interaction for cancer treatment. Inhibition of OCT4–PP1 interaction results in an increase of p-OCT4 (S236), leading to malignant cancer cell differentiation and growth retardation.

Despite its close relationship with cancer, clinical trials to treat cancer by targeting OCT4 have not been conducted. This is not due to side effects, because OCT4 does not function in normal adult stem cells [42]. A recent report has shown that perivascular cell-specific knockout of Oct4 inhibits angiogenesis [43], which leaves a therapeutic window for targeting OCT4. The main reason that there are no cancer treatment clinical trials targeting OCT4 is that there is no method to target OCT4 in a therapeutically effective way. In this study, we showed that a mimic of p-OCT4 (S236) induced GCT tumor differentiation and growth retardation in mouse xenograft experiments (Figure 4). Because the artificial inhibition of PP1 can increase p-OCT4 (S236) (Figure 5) and PP1 dephosphorylates OCT4 through direct physical interaction [34], a strategy to inhibit the physical interaction between PP1 and OCT4 would be an option for treating GCTs. Although developing drugs that target protein–protein interactions remains a challenge, several such drugs are already on the market [44]. In addition, since PP1 is ubiquitously expressed [45] and OCT4 is expressed in various solid tumors and plays a role in maintaining a malignant population [6,8,10], OCT4–PP1 interaction inhibitors, if developed, are likely to be applied to the treatment of various tumors in addition to GCTs (Figure 6).

## 4. Materials and Methods

### 4.1. Cell Culture and Chemicals

NCCIT (CRL-2073) and NTERA-2 (CRL-1973) were purchased from the American Type Culture Collection (Manassas, VA, USA) and maintained as described previously [37]. Cell lines were authenticated and regularly checked for *Mycoplasma* at the Genomics Core Facility (National Cancer Center, Gyeonggi-do, South Korea), as described previously [46]. Nocodazole (#487928) was purchased from Calbiochem (San Diego, CA, USA). Okadaic acid (#O8010), Dox (#9891), and crystal violet (#V5265) were purchased from Sigma–Aldrich (St. Louis, MO, USA). 

### 4.2. Western Blot

Western blot analysis was performed as described previously [47]. Anti-ACTB (A2228) antibody was purchased from Sigma–Aldrich; anti-Flag (#2368) was from Cell Signaling Technology (Danvers, MA, USA); anti-OCT4 (sc-5279) and anti-PP1γ (sc-6108) were from Santa Cruz Biotechnology (Santa Cruz, CA, USA). The anti-p-OCT4 (S236) antibody detecting human p-OCT4 (S236) [corresponding to murine p-Oct4(S229)] was identical to the anti-p-Oct4(S229) antibody described previously [34,35]. Goat anti-mouse IgG (#31430) was purchased from Pierce (Waltham, MA, USA). Goat anti-rabbit IgG (#1706515) and rabbit anti-goat IgG (H+L)-HRP conjugate (#1721034) were purchased from Bio-Rad (Hercules, CA, USA). 

### 4.3. Nocodazole Treatment and Flow Cytometry

NCCIT and NTERA-2 cells treated with nocodazole (50 ng/mL) for the indicated times were analyzed by propidium iodide staining (P4864; Sigma-Aldrich) and flow cytometry at the Flow Cytometry Core Facility (National Cancer Center) using FACSVerse (BD Biosciences, San Jose, CA, USA), as described previously [37].

### 4.4. Immunofluorescence and Confocal Microscopy

Immunofluorescence staining was performed as described previously [37]. Confocal images were obtained at the Microscopy Core Facility (National Cancer Center) using an LSM510 META and LSM 780 (Carl Zeiss, Jena, Germany).

### 4.5. Immunohistochemistry of a Patient Sample

Immunohistochemical staining was performed as described previously [41] using OCT4 and p-OCT4 (S236) antibodies. GCT patient samples were obtained from Ilsan Paik Hospital (Goyang, South Korea). The immunohistochemical staining for GCT patient samples was approved by the Ilsan Paik Hospital Institutional Review Board (2017-09-006-002), and IRB waived consent from patients. Two GCT patient samples were examined, and OCT4 and p-OCT4 (S236) were positive in both samples. An example of a sample that showed a stronger positivity is shown in Figure 1A. The patient sample that is shown is a 24-year-old male, pT2NxMx stage, diagnosed with mixed GCT (Teratoma: 50%; embryonal carcinoma: 40%; choriocarcinoma, biphasic: 10%).

### 4.6. Generation of Genetically Modified Cells

NCCIT and NTERA-2 Tet-on-shOCT4 cells expressing shRNA against OCT4 upon treatment with Dox were described previously [37]. NCCIT Tet-on-shPP1γ #1 and #2 cells were generated similarly. PP1γ-targeting shRNA sequences were as follows: shPP1γ #1 (GCG AAT TAT GCG ACC AAC TGA); shPP1γ #2 (ACA TTT GGT GCA GAA GTG GTT CT). The generation of OCT4 WT-rescued cells was described previously [37]. OCT4 S236D-rescued cells were generated similarly using the plasmid pCAG-Flag-BS-hOCT4 S236D, which was generated by the site-directed mutagenesis of pCAG-Flag-BS-hOCT4 WT [37]. Because shRNA against OCT4 (5′-TCATTCACTAAGGAAGGAATT-3′) was designed to target the 3′-untranslated region (UTR) of *POU5F1*, exogenously added constructs were not targeted by the shRNA. 

### 4.7. Clonogenic Assay and Tumor Sphere Formation Assay

The clonogenic assay (colony forming assay) and tumor sphere formation assay were performed as described previously [37,48].

### 4.8. AP Staining

AP staining assays were performed as described previously [37]. Briefly, naphthol/fast red violet solution was freshly prepared by mixing fast red violet (0.8 g/L in distilled water; Sigma), naphthol (4 mg/mL in 2 M 2-amino-2-methyl-1,3-propanediol buffer; Sigma), and distilled water at a ratio of 2:1:1. After the removal of the culture medium, cells were fixed with fixative solution [4% paraformaldehyde in phosphate-buffered saline (PBS)]. After rinsing with PBS, naphthol/fast red violet staining solution was added to each well and the plates were incubated in the dark at room temperature for 15 min. The wells were then rinsed with TBST (20 mM Tris-HCl, pH 7.4, 0.15 M NaCl, 0.05% Tween-20). The cells were covered with PBS to prevent drying, and the staining pattern was observed under an inverted light microscope.

### 4.9. RNA-Seq and Snalysis

Total RNA extracted from Mock, OCT4-depleted, OCT4 WT-rescued, and OCT4 S236D-rescued NCCIT cells were purified as described previously [37], and the preparation of RNA libraries and sequencing was performed by Macrogen (Seoul, South Korea) using the HiSeq 2500 sequencing platform (Illumina, San Diego, CA, USA). RNA-Seq data were deposited into the Gene Expression Omnibus database under the accession number GSE117521. DEGs were filtered for a fold-change cutoff of 2.0. Genes with < 1 fragment per kilobase of transcript per million mapped reads in all samples were excluded. Reactome analysis was performed using an online database (https://reactome.org/) with the DEGs as the source. Multi-Experiment Viewer 4.9.0 (mev.tm4.org) was used to graphically represent the values. Spearman’s rank-order correlation was used to determine the similarity among four samples with a significance level of 0.001. All analyses were performed using R version 3.6.2.

### 4.10. Animal Experiments

Animal experiments were performed as described previously [49]. Briefly, BALB/c-nu mice (female, 6–8 weeks old; Orient Bio, Seongnam, South Korea) were used to establish a tumor xenograft model. NTERA-2 Tet-on-shOCT4 OCT WT- and S236D-rescued cells (5.0 × 10^6^/each in opposite flanks) were inoculated subcutaneously. After 4–5 weeks, when the tumor sizes reached 50–100 mm^3^, the mice were randomly divided into two groups, a control group and a Dox group. Dox (1 mg/mL, #631311; Clontech Laboratories, Mountain View, CA, USA) was fed 5% sucrose drinking water. The primary tumor size and body weight were measured every week using calipers and a balance, respectively. The tumor volume was calculated using the formula V = (A × B^2^)/2, where V is the volume (mm^3^), A is the long diameter, and B is the short diameter. Mice were killed in a 7.5% CO_2_ chamber, and the tumors were harvested for further analysis. All mice were attended under specific pathogen-free conditions at the National Cancer Center Research Institute Animal Facility. This study was reviewed and approved by the Institutional Animal Care and Use Committee of the National Cancer Center Research Institute (NCC-17-401), an Association for Assessment and Accreditation of Laboratory Animal Care International-accredited facility.

### 4.11. Immunohistochemistry of Mouse Xenografts

The immunohistochemistry of mouse xenografts was done as described previously [49] at the Laboratory Animal Research Facility (National Cancer Center). For the histological analysis, the sections were stained with hematoxylin and eosin (H&E) according to the manufacturer’s protocol. H&E were purchased from Sigma–Aldrich (#H9627). Antibodies used were as follows: anti-OCT4 antibody (#ab181557, 1:50; Abcam, Cambridge, UK), anti-SOX2 (#3579, 1:100; Cell Signaling Technology), and anti-Ki67 (#ab15580, 1:300; Abcam).

### 4.12. Statistics

Statistical analyses were performed as previously reported [50]. All data are representative of at least three independent experiments, unless otherwise indicated. * *p* < 0.05, ** *p* < 0.01, *** *p* < 0.001 relative to control.

## 5. Conclusions

Inhibition of PP1 increases phosphorylation of OCT4 (S236), which in turn induces GCT differentiation by impairing the activity of OCT4. These results suggest that inhibition of the interaction between OCT4 and PP1 may be a therapeutic strategy for targeting OCT4 in various cancers.

## Supplementary Materials

The following are available online at https://www.mdpi.com/2072-6694/12/9/2601/s1. Table S1: Differentially expressed genes among the NCCIT cells harboring endogenous OCT4, depleted OCT4, OCT4 WT rescue, and OCT4 S236D rescue. Figure S1: Immunohistochemistry staining of the same GCT patient sample used in Figure 1A by anti-CKAP2 antibody.
