# Supplementary material for: Phosphorylation of OCT4 Serine 236 Inhibits Germ Cell Tumor Growth by Inducing Differentiation
Source: Cancers (Basel). 2020 Sep 11;12(9):2601. doi: 10.3390/cancers12092601 (PMC7565739; doi:10.3390/cancers12092601)

Figure S1: Immunohistochemistry staining of the same GCT patient sample used in Figure 1A by anti-CKAP2 antibody.

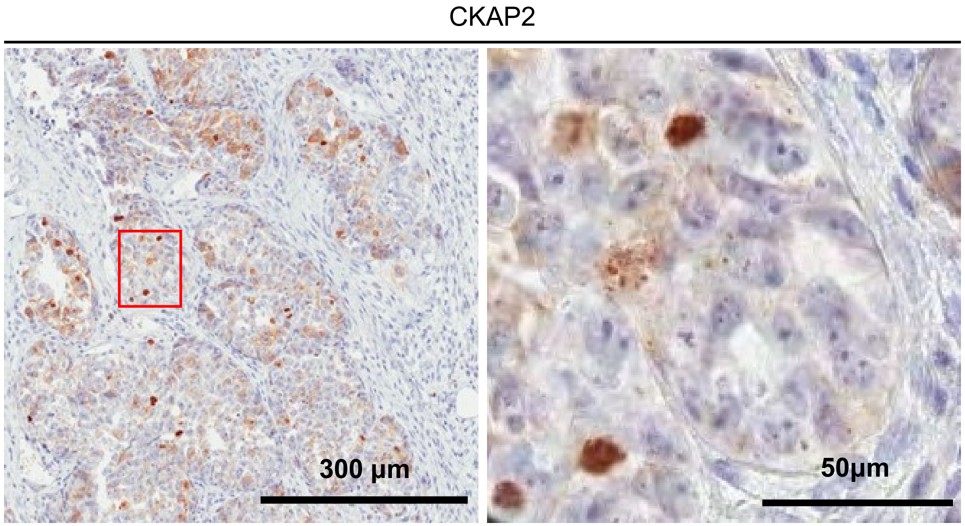

Supplement: Supplementary file 1 [file cancers-12-02601-s001.zip › cancers-914964-supplementary/Figure S1.pdf]
