# Supplementary material for: Phosphorylation of OCT4 Serine 236 Inhibits Germ Cell Tumor Growth by Inducing Differentiation
Source: Cancers (Basel). 2020 Sep 11;12(9):2601. doi: 10.3390/cancers12092601 (PMC7565739; doi:10.3390/cancers12092601)
Supplement: Supplementary file 1 [file cancers-12-02601-s001.zip › cancers-914964-supplementary/WB_wholeblot.pdf]

Figure 1B

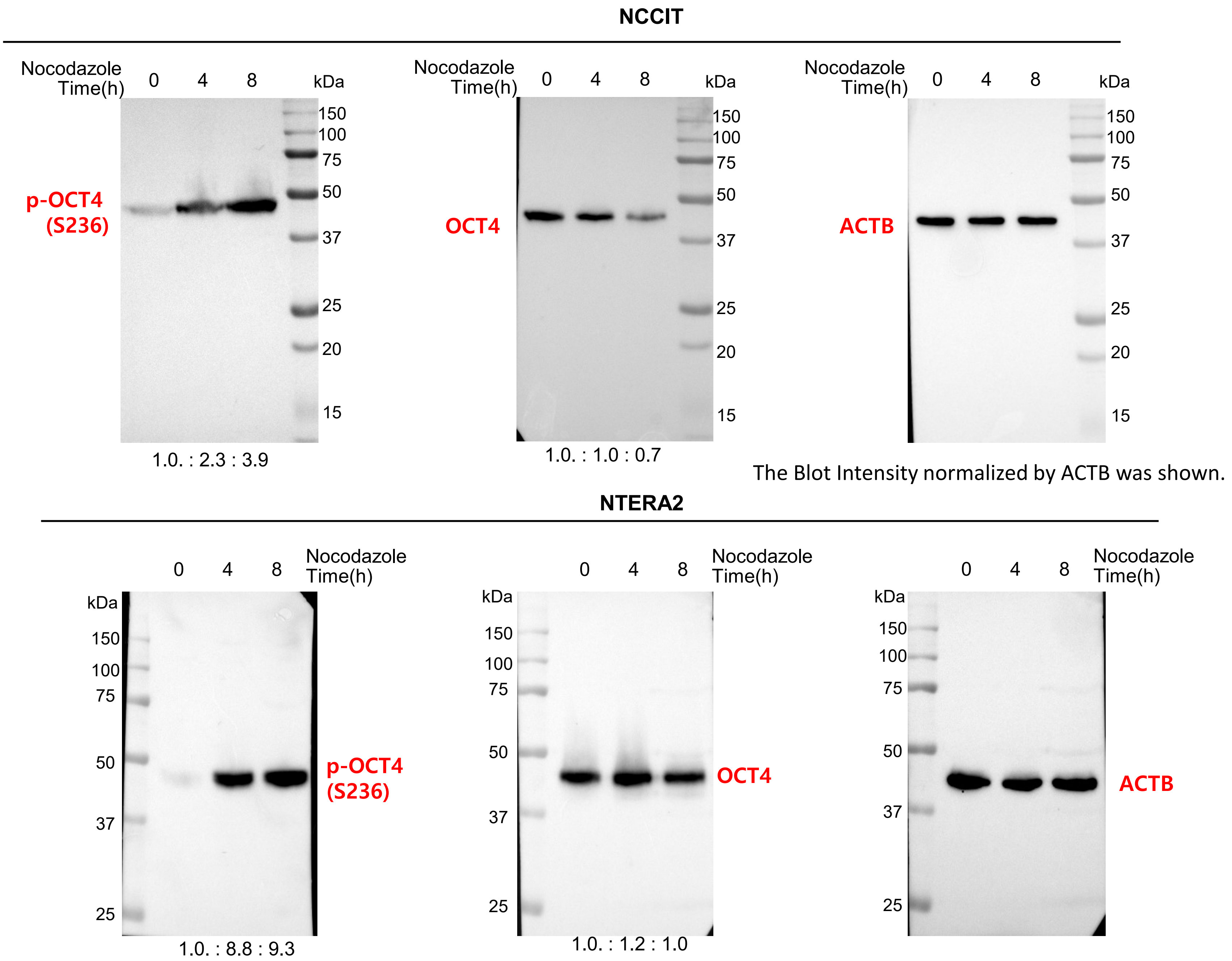
NCCIT
Nocodazole Time(h)
0
4
8
kDa
150
100
75
50
37
25
20
15
p-OCT4 (S236)
1.0. : 2.3 : 3.9
OCT4
1.0. : 1.0 : 0.7
ACTB
The Blot Intensity normalized by ACTB was shown.
NTERA2
p-OCT4 (S236)
1.0. : 8.8 : 9.3
OCT4
1.0. : 1.2 : 1.0
ACTB

Figure 1. B

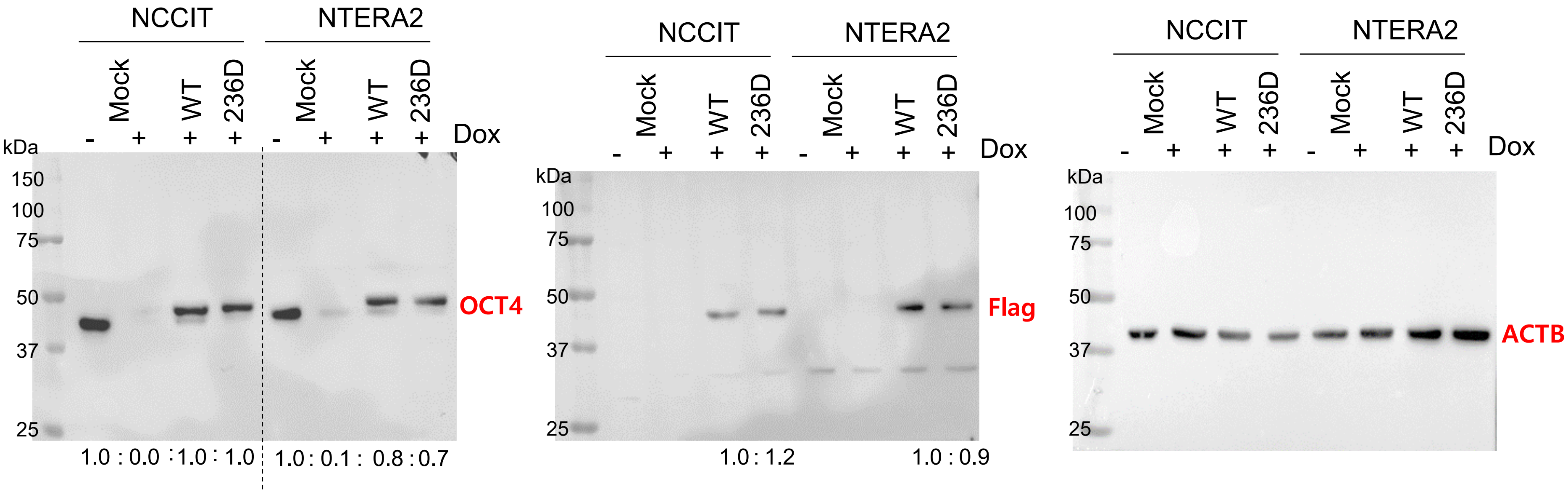
NCCIT
NTERA2
Mock
WT
236D
Dox
- + + + - + + +
kDa
150
100
75
50
37
25
OCT4
1.0 : 0.0 : 1.0 : 1.0
1.0 : 0.1 : 0.8 : 0.7
Flag
1.0 : 1.2
1.0 : 0.9
ACTB

Figure 1. C

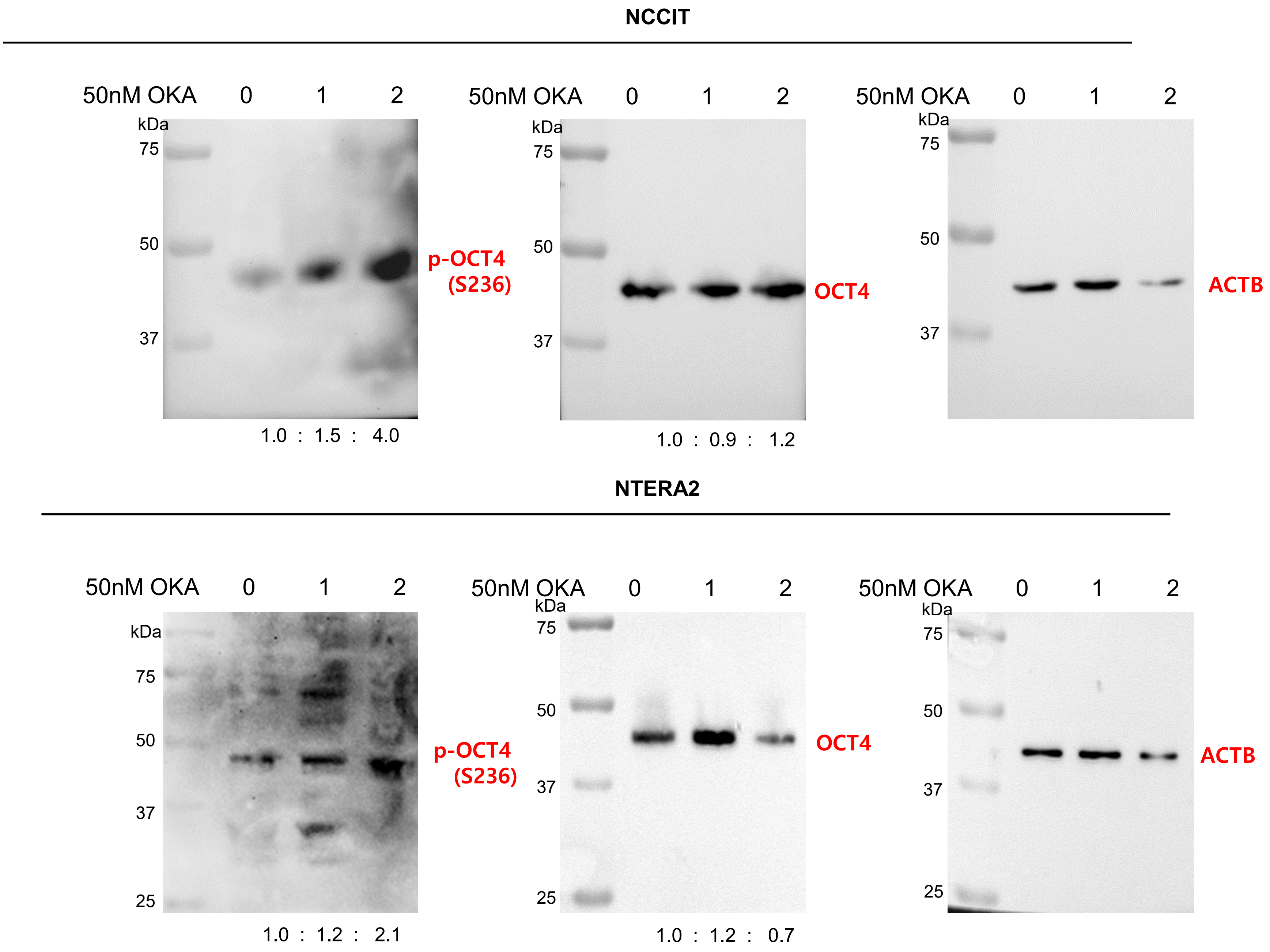
NCCIT
50nM OKA 0 1 2
kDa
75
50
37
p-OCT4 (S236)
1.0 : 1.5 : 4.0
50nM OKA 0 1 2
kDa
75
50
37
OCT4
1.0 : 0.9 : 1.2
50nM OKA 0 1 2
kDa
75
50
37
ACTB
NTERA2
50nM OKA 0 1 2
kDa
75
50
37
25
p-OCT4 (S236)
1.0 : 1.2 : 2.1
50nM OKA 0 1 2
kDa
75
50
37
25
OCT4
1.0 : 1.2 : 0.7
50nM OKA 0 1 2
kDa
75
50
37
25
ACTB

Figure 1. D

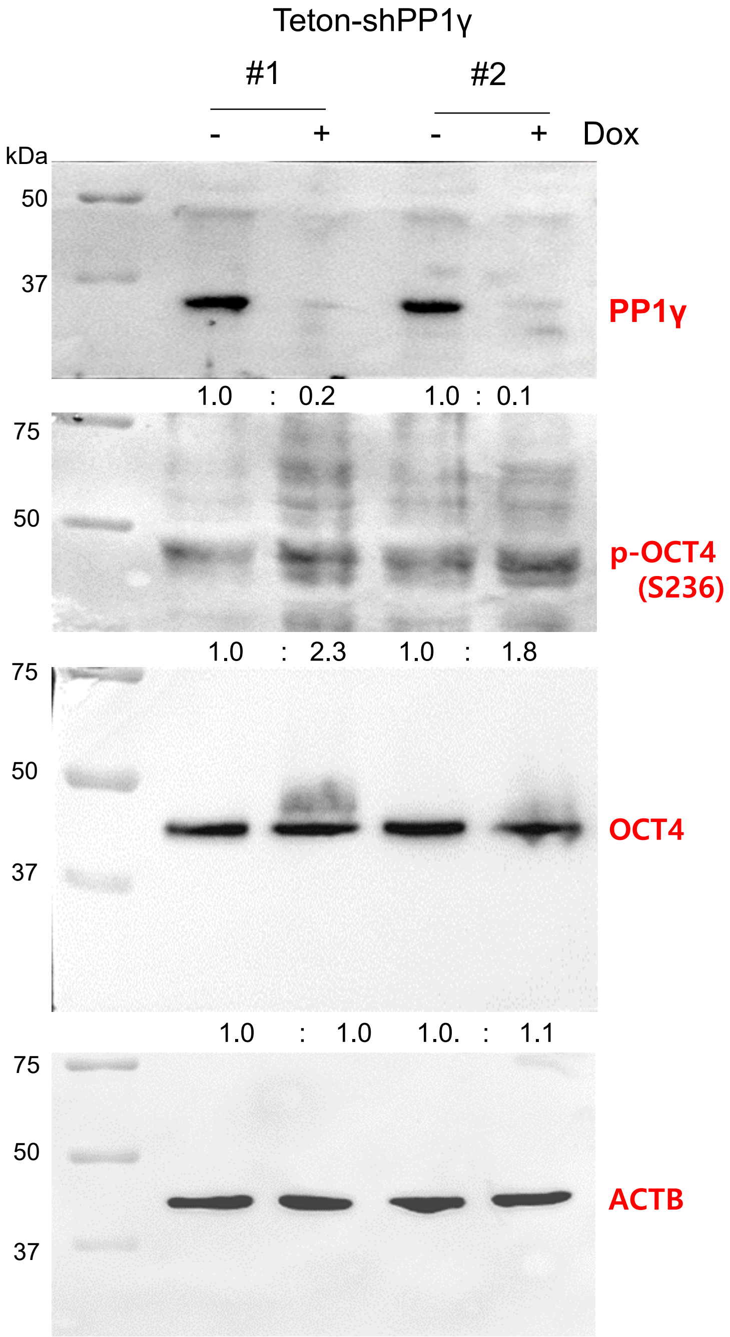

Teton-shPP1γ
#1
#2
- + - + Dox
kDa
50
37
PP1γ
1.0 : 0.2 1.0 : 0.1
75
50
p-OCT4
(S236)
1.0 : 2.3 1.0 : 1.8
75
50
37
OCT4
1.0 : 1.0 1.0. : 1.1
75
50
37
ACTB
